# Changes in DNA Methylation and Gene Expression of Insulin and Obesity-Related Gene *PIK3R1* after Roux-en-Y Gastric Bypass

**DOI:** 10.3390/ijms21124476

**Published:** 2020-06-24

**Authors:** Marcela A S Pinhel, Natália Y Noronha, Carolina F Nicoletti, Vanessa AB Pereira, Bruno AP de Oliveira, Cristiana Cortes-Oliveira, Wilson Salgado, Fernando Barbosa, Júlio S Marchini, Doroteia RS Souza, Carla B Nonino

**Affiliations:** 1 Laboratory of Nutrigenomics Studies, Health Science Department, Ribeirão Preto Medical School, Ribeirão Preto 14049-900, Brazil; marcelapinhel@yahoo.com.br (M.A.S.P.); natty.yumi@gmail.com (N.Y.N.); carol_nicolettif@yahoo.com.br (C.F.N.); vanessapereira@usp.br (V.A.B.P.); bruno_parenti@hotmail.com (B.A.P.d.O.); cristiana.cortes@outlook.com (C.C.-O.); wsalgado@fmrp.usp.br (W.S.J.); jsmarchi@fmrp.usp.br (J.S.M.); 2 Department of Molecular Biology, São José do Rio Preto Medical School, São José do Rio Preto 15090-000, Brazil; doroteiasouza@yahoo.com.br; 3 School of Pharmaceutical Sciences of Ribeirão Preto, Ribeirão Preto 14040-900, Brazil; fbarbosa@fcfrp.usp.br

**Keywords:** DNA methylation, insulin, obesity, microarray, gene expression, bariatric surgery, weight loss

## Abstract

Weight regulation and the magnitude of weight loss after a Roux-en-Y gastric bypass (RYGB) can be genetically determined. DNA methylation patterns and the expression of some genes can be altered after weight loss interventions, including RYGB. The present study aimed to evaluate how the gene expression and DNA methylation of *PIK3R1*, an obesity and insulin-related gene, change after RYGB. Blood samples were obtained from 13 women (35.9 ± 9.2 years) with severe obesity before and six months after surgical procedure. Whole blood transcriptome and epigenomic patterns were assessed by microarray-based, genome-wide technologies. A total of 1966 differentially expressed genes were identified in the pre- and postoperative periods of RYGB. From these, we observed that genes involved in obesity and insulin pathways were upregulated after surgery. Then, the *PIK3R1* gene was selected for further RT-qPCR analysis and cytosine-guanine nucleotide (CpG) sites methylation evaluation. We observed that the *PI3KR1* gene was upregulated, and six DNA methylation CpG sites were differently methylated after bariatric surgery. In conclusion, we found that RYGB upregulates genes involved in obesity and insulin pathways.

## 1. Introduction

The worldwide increase in the obesity epidemic is a consequence of an obesogenic environment characterized by an imbalance between energy intake and expenditure [1]. Such environmental factors have been linked to a rapid weight gain; however, genetic alterations may also contribute substantially to obesity [2,3]. Consequently, obesity can result from genetic and epigenetic factors that influence body weight maintenance [4], in addition to an unbalanced glucose and insulin homeostasis. Different metabolic pathways affect the development of this complex disease, namely energy expenditure regulation, adipocyte differentiation, adipose tissue accumulation, and pathways related to eating behavior [5].

The Roux-en-Y gastric bypass (RYGB)—the most common surgical procedure to treat obesity after the failure of conventional treatments [6]—can also improve comorbidities related to obesity [7]. Furthermore, given the role of genetics in weight regulation, the magnitude of weight loss after surgery can also be genetically determined [8]. As a consequence, numerous biological pathways may be altered in obese patients, and bariatric surgery could restore the normal functions of regulatory networks [9].

Epigenetic modifications may also indicate critical relationships between genetic, environmental, and lifestyle factors. DNA methylation patterns are currently well characterized for chronic diseases; methylation is characterized by the presence of methyl (−CH3) groups at cytosine-guanine nucleotide (CpG) sites. However, it remains unclear how DNA methylation influences gene transcription, obesity and its related comorbidities [10].

The phosphatidylinositol 3-kinase (PIK3) signaling pathway plays a vital role in insulin metabolism. Several reports show that the canonical pathway PIK3/AKT is disrupted in obese subjects with and without diabetes compared with non-obese subjects [11]. PIK3 has both catalytic (PIK3CA and PIK3CD) and regulatory subunits (PIK3R1), being the last one critical for obesity studies since its expression is linked to insulin metabolism and initiation of inflammation processes [12]. 

Currently, the patterns of individual CpG sites of *PIK3R1* before and after bariatric surgery remain uncharacterized. Thus, in the present study, we aimed to determine the impacts of RYGB on the expression and DNA methylation patterns of the *PIK3R1* gene. We chose to use whole blood as the target tissue as it reflects the systemic response to metabolic alterations and is an essential biological tissue for biomarker discovery [13]. As far as we know this was the first study to analyze the pattern of individual CpG sites of *PIK3R1* before and after bariatric surgery.

## 2. Results

### 2.1. Anthropometrics Data and Biochemical Profile

The mean preoperative weight of the 13 investigated female patients was 115.3 ± 19.4 kg (BMI: 42.5 ± 7 kg/m²), and a 6-month weight loss corresponded to 24.7% of initial weight and 54.4% of excess body weight (EBW). Four patients (12.5%) had type 2 diabetes in the preoperative period and were taking metformin as the primary treatment. After surgery, we observed that blood glucose concentration decreased from 93.3 ± 13.5 to 83.6 ± 28.4 mg/dL. Table 1 shows the anthropometric data and biochemical profile of obese patients before and six months after RYGB. Data for comorbidities, life habits, and family history of obesity were previously reported by our group [14].

### 2.2. Gene Expression 

The comparison of gene expression profiles in obese patients before and after RYGB revealed that 1188 genes were upregulated (Supplementary File 1). Gene Ontology (GO) enrichment analysis showed that 13 genes were significantly involved with the onset of obesity and insulin-related pathways (*p*-value < 0.05). Then we selected genes with *p*-values ≤0.05 and fold change cutoff of ≥2 are presented in Table 2. The description of the pipeline for GO calculations can be accessed at http://geneontology.org/docs/go-enrichment-analysis/.

**Figure 1:**
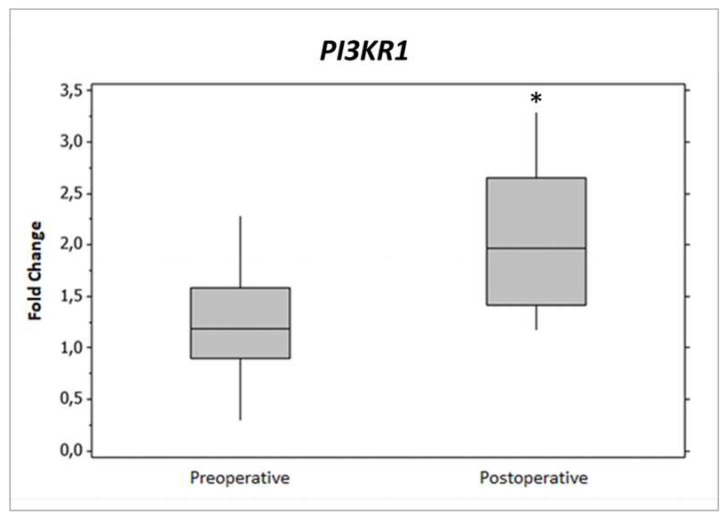
Relative gene expression of phosphoinositide-3-kinase regulatory subunit 1 (*PIK3R1*) in pre- and postoperative periods. Both periods were composed of 13 patients. The fold change was calculated with the equation 2^−∆∆*C*t^. * *p* < 0.05.

*PIK3R1* gene expression was significantly enriched in both lists (*p* < 0.05) from Table 2, because it was chosen to be validated and analyzed more deeply by DNA methylation. *PIK3R1* gene expression was verified by quantitative RT-qPCR (Figure 1).

We compared results for *PIK3R1* gene expression, including and excluding the data from two patients that had blood glucose values above the recommended. Since we did not find any difference in such comparison, the patients were kept for further analyses.

### 2.3. CpG Methylation of PIK3R1

**Figure 2:**
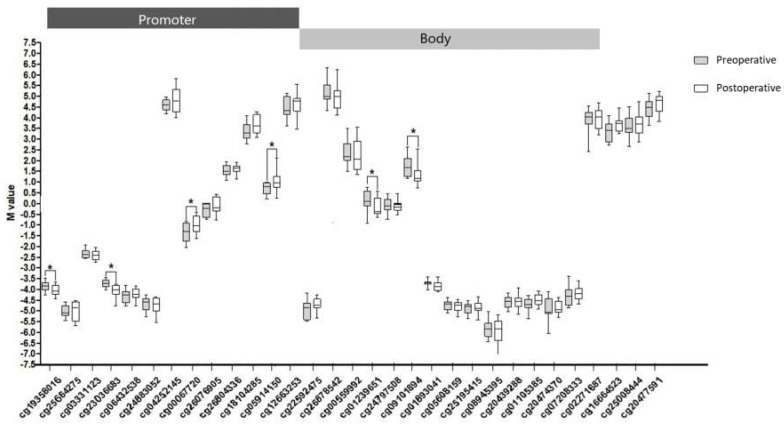
M values of evaluated CpG sites in promoter and body regions of *PI3KR1* gene in pre- and postoperative periods of RYGB. * *p* < 0.05 shows CGs statistically significant in patients.

After quality control checks, we obtained 410.586 valid probes. Using iMETHYL [15], we found that 31 probes were mapped to the *PIK3R1* gene. M values for all probes are shown in Figure 2. When comparing the data before and after RYGB, we found six differentially methylated CpG sites (cg19358016, cg23036683, cg00067720, cg05914150, cg01239651, and cg09101894). These probes were primarily hypomethylated in patients after surgery, and the majority of them were located in CpGs of the promoter region (TSS 200 and 1500) and 5’ UTR of *PIK3R1* gene.

### 2.4. Relationship between CpG Methylation and PI3KR1 Gene Expression

**Figure 3:**
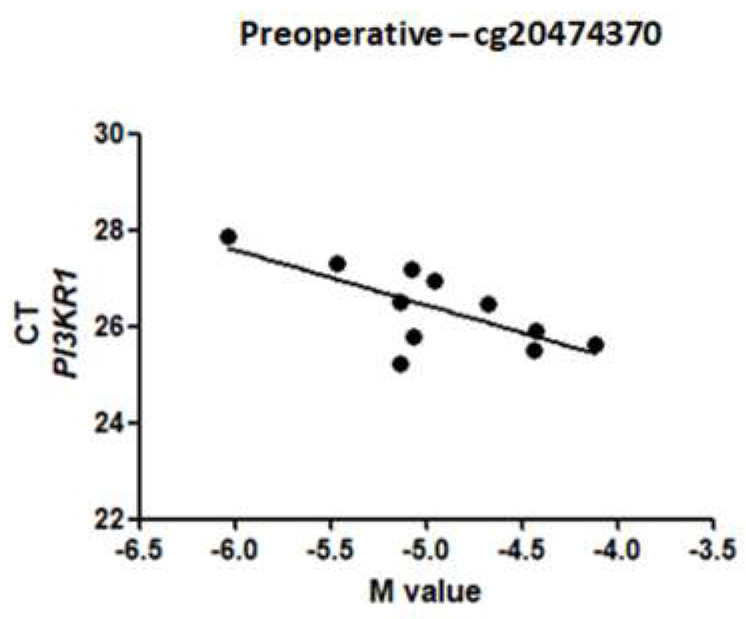
Correlation between gene expression data (RT-qPCR) and the M value in the site cg20474370 of *PI3KR1* in before RYGB (*r* = −0.709, *p* < 0.01). CT: cycle threshold.

After stepwise linear regressions, three CpG sites were associated with RT-qPCR values (*p* < 0.005): cg22592475, cg26878542, and cg20474370. However, only cg20474370 strongly correlated with gene expression in the preoperative group (Pearson correlation, *r* = −0.709, *p* = 0.001) (Figure 3). To build a multivariable linear regression model, we included the cycle threshold (CT) gene expression data values and the M values for DNA methylation. Moreover, the model was adjusted by the endogenous control expression data. The β values, multiple *r*², and *p*-values are shown in Table 3. The β values represent the direction of the association and *r*² the percentage of explanation of the model.

## 3. Discussion

We have longitudinally determined whether surgical weight loss alters the transcriptome of leucocytes in obese women. In this report, we show that genes involved in obesity and insulin pathways are upregulated after RYGB. Our results are in keeping with the findings from a pilot study showing that the expression levels of 200 transcripts linked to obesity and type 2 diabetes change significantly after bariatric surgery [16]. Genetic and environmental factors alter signaling and enzymatic networks that control glucose and lipid metabolism, resulting in obesity and diabetes development and maintenance [17]. Here, we show that *PIK3R1* expression levels have increased significantly after RYGB. 

**Figure 4:**
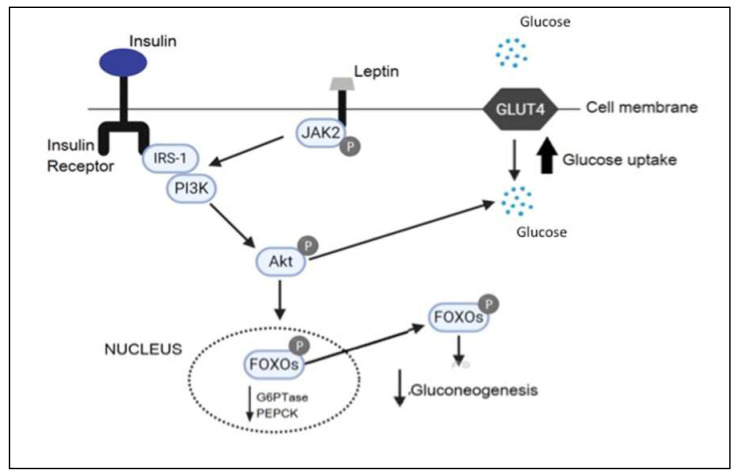
RYGB improves insulin and leptin resistance with consequent IRS-1 phosphorylation to initiate a signaling cascade that involves PIK3. PIK3 activation leads to Akt downstream signaling. The phosphorylation of IRS-1 triggered by PIK3 allows glucose to enter the cell improving blood glucose levels.

PIK3 signaling is one of the main pathways by which leptin and insulin alter neuronal excitability in the hypothalamus [18]. The deletion of *PIK3* in POMC neurons suppresses food intake [19]. Moreover, PIK3 signaling partially modulates the action of leptin and insulin on food intake [20]. On the other hand, peripheral inflammation due to obesity is a key component for developing type 2 diabetes in these patients. The excess of adipose tissue increases secretion of inflammatory cytokines such as TNF-alpha and IL-6 that inhibit phosphorylation of insulin receptors (IRS-1), promoting insulin resistance [21]. Therefore, JAK2 autophosphorylation leads to the recruitment and phosphorylation of IRS-1, which then recruits PIK3 and activates downstream signals [22]. For this reason, we hypothesized that reduced adipose tissue after RYGB improves insulin resistance and activates PIK3. This might decrease weight and increase hyperglycemia through activation of FOXO1 and GLUT4 (Figure 4).

We selected the *PIK3R1* gene for an in-depth analysis since it is critical for obesity, insulin resistance, and may also be affected by DNA methylation [23]. Some authors suggest that activation of PIK3 can affect the response of cells to energy balance modulation; however, this mechanism is still not fully understood [24]. The downregulation of several genes has already been reported to initiate an inflammatory response in the adipose tissue, decreasing insulin sensitivity [12] (p. 2). We hypothesized that DNA methylation would be a critical factor in explaining the increased gene expression in the postoperative period.

After bariatric surgery, several DNA methylation sites were differentially methylated, and the majority were hypomethylated. M-values close to 0 indicate that the site is half methylated, positive M-values indicate that the site is hypermethylated, and negative values indicate the opposite [25]. A strong negative correlation was observed between cg20474370 methylation and *PIK3R1* expression data in the preoperative period. Some other weak and medium (<0.6) correlations were found, but those correlations changed after surgery. This result suggests that epigenetic triggers are different in obese patients after bariatric surgery. 

The presence of significant metabolic pathway changes in the postoperative period may per se be an epigenetic stimulus to change the methylome. Zuo et al. (2012) suggested that the activation of *PIK3* might influence downstream epigenetic control in cultured cancer cells [26]. This statement suggests that *PIK3* may both be affected and promote epigenetic alterations. Using a multivariate linear regression model, we found that the methylation levels of cg20474370 could explain around 70% of *PIK3R1* gene expression when corrected using endogenous controls in the preoperative period. Nevertheless, in the postoperative period, correlations were weaker, suggesting that epigenetic mechanisms that control *PIK3R1* gene expression are altered after surgery. The punctual effects of DNA methylation are still unclear and should be further examined. 

We found a weak correlation between *PIK3R1* gene expression and methylation. However, one cannot disregard that DNA methylation has effects on *PIK3R1* expression since we observed differentially methylated probes located at the promoter and 5′ UTR regions. These regions control gene expression and are part of the traditional DNA methylation paradox [27]. Indeed, studies have verified that PIK3 expression is influenced by DNA methylation [28]. It is known that methylation changes in CpGs present in the gene body can promote biological effects [27] (p. 6). Thus, experiments with higher resolution of CpG sites may increase the accuracy of our findings. Similarly, next-generation sequencing experiments may provide a better overview of the transcriptomic changes after RYGB. 

## 4. Materials and Methods

We investigated 13 women with severe obesity (Body Mass Index, BMI > 40 kg/m^2^) before and six months after RYGB. Patients who were not submitted to the standard surgical technique, patients who lost follow-up with the multidisciplinary team, pregnant women, and patients with thyroid disease, cancer, and psychiatric disorders were excluded. Anthropometric measurements (weight, height, and BMI) and blood samples were obtained before and after surgery. All subjects signed an informed consent term before participating in the study. The study was conducted following the Declaration of Helsinki, and the protocol was approved by the Ethics Committee of the Clinical Hospital of Ribeirão Preto Medical School (Certificate of Presentation for Ethical Consideration—CAAE: 18973913.0.0000.5440, approval date: 14 June 2016).

### 4.1. RNA Isolation

Total RNA was extracted and purified from blood cells using the phenol-chloroform extraction method. The quality and concentration of RNA were determined with the Agilent 2100 Bioanalyzer and the Agilent RNA 6000 Nano Kit (Agilent, Amstelveen, Netherlands). To evaluate gene expression, samples with RNA integrity number (RIN) higher than 7.0 were used in the analysis.

### 4.2. Gene Expression Analysis

RNA amplification and labeling were performed with an Epicenter Target Amp Nano-g Biotin a-RNA kit (Labeling kit for Illumina^®^) that produced amplified biotinylated cRNAs with an input of 500 ng of total RNA. The hybridization protocol used 750 ng cRNA on HumanHT-12_v4 slides in specific buffer at 58 °C for 16 h, which was followed by washings and streptavidin-Cy3 staining. The slides were scanned with the Illumina Bead Array Reader (iSCAN^®^) on green channel only. The global gene expression analysis of these patients was previously reported by our group [14].

The first-line check and background correction were performed with the GenomeStudio Software (Illumina^®^). Samples with less than 6000 significantly detected probes (detection *p*-value < 0.01) were excluded from further analyses. Differentially expressed genes obtained between pre- and post-RYGB were identified after a cutoff for fold change >2 in gene expression and of a t-test *p*-value of less than 0.05. These *p*-values were corrected for multiple comparisons by using a false discovery rate (FDR) of 0.05 [11] (p. 2). The microarray data were deposited in the Gene Expression Omnibus database under access number GSE83223 (https://www.ncbi.nlm.nih.gov/geo/query/acc.cgi?acc=GSE83223). 

### 4.3. Validation Analysis

Microarray gene expression was confirmed through RT-qPCR using TaqMan^®^Gene Expression Assays (Thermo Fisher, Foster City, CA) for three genes (*PIK3R1*, *GAPDH* and *ACTINB*). First, total RNA was reverse transcribed into cDNA using the High Capacity cDNA Reverse Transcription Kit (ThermoFisher). The TaqMan technology suitably quantifies relative gene expression [13] (p. 2). *GAPDH* and *ACTINB* were used as reference genes since their expression does not vary, as corroborated by the data obtained from the microarray analysis. Triplicates and positive and negative controls were included in all reactions. Table 4 presents the details of expression assays used. The final reaction was 20 uL, and each reaction was used as an input of 50 ng of total RNA.

### 4.4. DNA Extraction

The blood for DNA methylation analysis was collected after 12 hours of fasting. DNA was extracted from peripheral blood leukocytes and used to hybridize 450k BeadChip according to the manufacturer’s instructions.

### 4.5. DNA Methylation Analysis

The methylation data were analyzed using the Infinium Human Methylation 450k BeadChip array following Illumina Methylation HD BeadChip protocol, as previously described by our group in the same set of patients [29]. The Beadchips were scanned with the Illumina IScanSQ system, and the image intensities were extracted with the Genome Studio 2.0. The methylation level was expressed as a beta (β) value—calculated as the intensity of the methylated channel divided by the total intensity β values range from 0 (unmethylated) to 1 (fully methylated). β values can be broadly interpreted as the percentage of CpG methylation.

Quality control and normalization of β values (BMIQ method) were performed using the Champ package for R. We filtered out probes with *p*-values above 0.01 and beadcount <3 in at least 5%, confounding starts (NoCG, SNPS, Multhit, and XY). For statistical analyses, β values were transformed in M values using the equation M value = log2 (β/1 − β), as described elsewhere [30]. Comparison of β values and M values methods for quantifying methylation levels by microarray analysis were performed. Further, we mapped the CpGs of interest located in genomic regions of the three targets validated by RT-qPCR using the integrative multi-omics database iMETHYL [12] (p. 2).

### 4.6. Statistical Analysis

Statistical analyses were performed using Statistical Package for the Social Sciences- SPSS v.20. The Shapiro-Wilk test was used to verify if clinical data were normally distributed, and values were compared using parametric or equivalent non-parametric tests. *p*-values <0.05 were considered as statistically significant. For RT-qPCR data, 2^−ΔΔCt^ was calculated based on Livak recommendations, geometric average for *GAPDH*, and *ACTINB* were used as endogenous control [14]. Eutrophic women were used as reference groups for the analyses. To investigate the relationship between gene expression and DNA methylation data, we used M values. Individual linear regressions were conducted to check associations before building multivariable linear regression models [31]. All CpG sites significantly associated with gene expression data (*p*-value < 0.05) were included in the final model. Glycemia was tested but did not change r^2^ considerably; thus, endogenous control genes were used to adjust the final model. The linear regression models were conducted using the lm( ) function in R v.3.5.2 (RStudio, Inc., Boston, MA, USA). 

## 5. Conclusions

The weight loss induced by bariatric surgery can alter the expression of genes involved in insulin metabolism and obesity pathways. Overexpression of genes related to these pathways may be associated with the improvement of glucose metabolism, and DNA methylation impacts it partially. 

## Figures and Tables

**Table 1:** Anthropometric data and biochemical profile of obese patients in preoperative and postoperative (six months after Roux-en-Y gastric bypass (RYGB)).

Variables	Preoperative	Postoperative
N	13	13
Age (years)	35.9 ± 9.2	36.2 ± 9.3
Weight (kg)	115.3 ± 19.4	85.3 ± 13.8 ^a^
BMI (kg/m²)	42.5 ± 7.0	31.8 ± 5.4 ^a^
TC (mg/dL)	177.4 ± 32.7	152.5 ± 28.4 ^a^
HDL-C (mg/dL)	41.3 ± 6.0	44.3 ± 8.1
LDL-C (mg/dL)	112.4 ± 27.6	88.6 ± 23.0 ^a^
TG (mg/dL)	129.7 ± 42.7	75.0 ± 27.0 ^a^
Glucose (mg/dL)	93.3 ± 13.5	83.6 ± 28.4 ^a^

Mean ± standard deviation; BMI: body mass index; %EWL: excess weight loss; TC: total cholesterol; HDL-C: high-density lipoprotein cholesterol; LDL-C: low-density lipoprotein cholesterol; TG: triglycerides; a: *p*-value < 0.05 compared to preoperative.

**Table 2:** Upregulated genes after six months of RYGB that are involved in the onset of obesity and insulin pathway (*p*-value < 0.05). The fold change results were derived from the microarray assay.

Symbol	Gene Name	Fold Change *
Obesity		
*EPS8*	Epidermal growth factor receptor pathway substrate 8	5.57 × 10^6^
*PTPN2*	Protein tyrosine phosphatase, non-receptor type 2	2.79 × 10^6^
*ACTR3*	ARP3 actin-related protein 3 homolog	2.32 × 10^6^
*RPS6KA3*	Ribosomal protein S6 kinase	2.45 × 10^6^
*MAPK9*	Mitogen-activated protein kinase 9	2.26 × 10^6^
*RAP1B*	RAP1B member of RAS oncogene family	5.51 × 10^6^
*PLA2G4A*	Phospholipase A2	9.65 × 10^6^
*PIK3R1*	Phosphoinositide-3-kinase, regulatory subunit 1	6.55 × 10^6^
**Insulin Pathway**		
*EIF4E*	Eukaryotic translation initiation factor 4E	3.76 × 10^6^
*IKBKB*	Inhibitor of kappa light polypeptide gene enhancer in B-cells kinase beta	3.90 × 10^6^
*PHKB*	Phosphorylase kinase beta	4.13 × 10^6^
*MAPK9*	Mitogen-activated protein kinase 9	2.26 × 10^6^
*PPP1CB*	Protein phosphatase 1, catalytic subunit, beta isozyme	2.19 × 10^6^
*SOCS4*	Suppressor of cytokine signaling 4	4.13 × 10^6^
*PIK3R1*	Phosphoinositide-3-kinase, regulatory subunit 1 alpha	6.55 × 10^6^

* *p*-value < 0.05.

**Table 3:** Multiple linear regression showing the contribution of cg20474370 in gene expression.

Gene Symbol (Gene ID)	Site	Gene Region	β	Adjusted Multiple *r*²	*p*-Value
*PI3KR1* (ENSG00000145675)	cg20474370	Body	−0.59	0.70	0.003

β: regression coefficient, *r*²: coefficient of determination. Regression was adjusted by endogenous control (RT-qPCR data).

**Table 4:** RT-qPCR TaqMan^®^Gene Expression Assays.

Gene	ID (Gene Expression Assay)	Fragment (pb)
*PIK3R1*	Hs00933163	82
*GAPDH*	Hs99999905	122
*ACTINB*	Hs99999903	171

## Supplementary Materials

Supplementary materials can be found at https://www.mdpi.com/1422-0067/21/12/4476/s1.

## Abbreviations

RYGB	Roux-en-Y Gastric Bypass
CAAE	Certificate of Presentation for Ethical Consideration
BMI	Body Mass Index
RIN	RNA Integrity Number
FDR	False Discovery Rate
GO	Gene Ontology
